# Prefixed-Threshold Real-Time Selection for Free-Space Sending-or-Not Twin-Field Quantum Key Distribution

**DOI:** 10.3390/e24030344

**Published:** 2022-02-27

**Authors:** Yang Yu, Rui Xu, Le Wang, Qianping Mao, Shengmei Zhao

**Affiliations:** 1 Institute of Signal Processing Transmission, Nanjing University of Posts and Telecommunications (NUPT), Nanjing 210003, China; 2021010209@njupt.edu.cn (Y.Y.); 1318014614@njupt.edu.cn (R.X.); lewang@njupt.edu.cn (L.W.); 2 College of Computer Science and Technology, Nanjing Tech University, Nanjing 211816, China; maoqp1@njtech.edu.cn; 3 Key Laboratory of Broadband Wireless Communication and Sensor Network Technology, Ministry of Education, Nanjing 210003, China

**Keywords:** sending-or-not twin-field quantum key distribution, free-space quantum key distribution, prefixed-threshold real-time selection, observable model

## Abstract

As a variant of the twin-field quantum key distribution (TF-QKD), the sending-or-not twin-field quantum key distribution (SNS TF-QKD) is famous for its higher tolerance of misalignment error, in addition to the capacity of surpassing the rate–distance limit. Importantly, the free-space SNS TF-QKD will guarantee the security of the communications between mobile parties. In the paper, we first discuss the influence of atmospheric turbulence (AT) on the channel transmittance characterized by the probability distribution of the transmission coefficient (PDTC). Then, we present a method called prefixed-threshold real-time selection (P-RTS) to mitigate the interference of AT on the free-space SNS TF-QKD. The simulations of the free-space SNS TF-QKD with and without P-RTS are both given for comparison. The results showed that it is possible to share the secure key by using the free-space SNS TF-QKD. Simultaneously, the P-RTS method can make the free-space SNS TF-QKD achieve better and more stable performance at a short distance.

## 1. Introduction

Even with a malicious third party, Eve, the quantum key distribution (QKD) can provide theoretically secure secret keys between two legitimate users, Alice and Bob, based on the mechanics of quantum physics. The first QKD protocol named BB84 was proposed in 1984 by Bennett and Brassard [1]. Ever since then, plenty of QKD protocols, such as decoy-state QKD protocols [2,3,4], measurement-device-independent quantum key distribution (MDI-QKD) protocols [5,6,7,8,9,10], round-robin differential-phase-shift quantum key distribution (RRDPS-QKD) protocols [11,12,13], etc., have been proposed to overcome the obstacles in the QKD’s development.

When the TF-QKD was announced in 2018 [14], it broke the PLOB bound [15], held for all the previous QKD protocols without quantum repeaters. This work altered the relationship between the secure key rate (SKR) and the channel loss from linear dependence to square dependence. It was shown that the transmission distance was greatly enlarged in comparison to those existing protocols under the same circumstance. Inspired by the TF-QKD, a series of the twin-field-like protocols was presented [16,17,18,19], where the sending-or-not (SNS) TF-QKD stands out for its high tolerance of misalignment error in the QKD procedure [20,21,22,23,24,25,26].

Recently, the QKD through free-space channels has become a hot topic, since it allows the users to communicate on mobile platforms, which is inconvenient for optical fiber quantum communication. It became more attractive when the Micius quantum experiment science satellite was launched [27,28]. Furthermore, it allows users to realize not only satellite-to-ground communications, but also communications on any mobile platforms via water and air. Compared with the other TF-QKD, the SNS TF-QKD has higher tolerance of misalignment error. There has already been an experimental demonstration of the SNS TF-QKD over a 509 km-long ultra-low loss optical fiber [29], which provided the implementation feasibility of the SNS TF-QKD in wire communication. However, a major obstacle of the free-space QKD is the interference caused by atmospheric turbulence (AT), where the channel transmittance is varied with time, not as the channel transmittance as a constant in fiber communications.

To deal with the channel transmittances fluctuating with time, some methods have been proposed [30,31]. One is to monitor the real-time channel transmittances, optimize a threshold according to the recorded transmittance during the post-processing, and use it to discard all the rounds whose the transmittance is less than a threshold. However, this demands storage requirements for Bob and optimization calculation. In 2018, Lo et al. modified the idea and proposed a new method, called prefixed-threshold real-time selection (P-RTS), to prefix the threshold even before the experiment begins and used it to select the real-time signals [32]. In 2019, they further extended the idea from the one-way QKD protocol to a two-way QKD protocol [33]. The sending-or-not (SNS) TF-QKD is in principle a two-way QKD protocol, which makes the method applicable to it.

In this paper, we first present the mathematical model of the free-space channel and the characteristic of the channel transmittances. Then, we demonstrate the observable model and applied it to the SNS TF-QKD. After that, the function of the asymmetric SNS TF-QKD protocol versus channel transmittances $\eta_{a}$ and $\eta_{b}$ is presented. Furthermore, the SKR function $R(\eta_{a},\eta_{b})$ was plotted out in order to determine the domain where the signals were selected. Finally, some numerical simulations are given based on the observable model to analyze the performance of the free-space SNS TF-QKD under AT, with and without the P-RTS method.

## 2. Prefixed-Threshold Selection for the Free-Space SNS TF-QKD

### 2.1. Free-Space Channel Model

The channel transmittance η, which fluctuates with time, is one of the free-space channel’s major characteristics, which is caused by AT. Such a characteristic can lead to a time-varying SKR. That is, the SKR may be low at some time points. It was discussed how the phase-matching quantum key distribution’s SKR decreases greatly in the free space [34]. One solution is post-selecting signals based on the monitored channel transmittance. Therefore, the model of the free-space channel is required in order to mathematically describe the SKR performance of the free-space protocols with and without transmittance post-selection.

The time-varying transmittance η follows a probability distribution of the transmission coefficient (PDTC). There exist multiple models to describe the PDTC. Among those models, the log-normal model is widely used [35], which can be described as,

(1) $$p_{\eta_{0},\sigma }\left(\eta \right)=\frac{1}{\sqrt{2\pi }\sigma \eta }e^{-\frac{{\left[ln\left(\frac{\eta }{\eta_{0}}\right)+\frac{1}{2}\sigma^{2}\right]}^{2}}{2\sigma^{2}}}$$

where *p* denotes the probability density, $\eta_{0}$ represents the expected atmospheric transmittance, and σ is the variance determined by the amount of turbulence and usually takes a value between zero and one. $\eta_{0}$ and σ are the intrinsic parameters of the channel and, thus, contain all the information of the PDTC.

After applying the post-selection of η with a threshold $\eta_{T}$ and using the expected value formula for a truncated distribution, one can obtain a higher mean transmittance among the selected signals as,

(2) $$\langle \eta \rangle =\frac{\int_{\eta_{T}}^{1}\eta p_{\eta_{0},\sigma }\left(\eta \right)d\eta }{\int_{\eta_{T}}^{1}p_{\eta_{0},\sigma }\left(\eta \right)d\eta }.$$

Assume the two channels in the SNS TF-QKD protocol (Alice to Charlie and Bob to Charlie) are free-space channels and the fluctuations of their transmittances can be described by the log-normal model. Owing to the assumption that the two channels are separate and independent, the joint PDTC can be written as [33],

(3) $$p_{AB}\left(\eta_{a},\eta_{b}\right)=p_{\eta_{A_{0}},\sigma_{A}}\left(\eta_{a}\right)\cdot p_{\eta_{B_{0}},\sigma_{B}}\left(\eta_{b}\right),$$

where $\left(\eta_{A_{0}},\sigma_{A}\right)$ and $\left(\eta_{B_{0}},\sigma_{B}\right)$ are the condition parameters of the two channels, respectively. The two variables $\eta_{a}$ and $\eta_{b}$ form a plane on which the joint PDTC is defined.

Since Charlie can post-select signals by observing both channels’ transmittances, the threshold is actually extended to two dimensions, namely an area in the plane defined by $\left(\eta_{a},\eta_{b}\right)$ [33]. Thus, a double-integral should be performed in order to obtain the excepted values of the transmittances $\eta_{a}$ or $\eta_{b}$, that is,

(4) $$\langle \eta_{x}\rangle =\frac{\int \int_{\Omega }\eta_{x}p_{AB}\left(\eta_{a},\eta_{b}\right)d\eta_{a}d\eta_{b}}{\int \int_{\Omega }p_{AB}\left(\eta_{a},\eta_{b}\right)d\eta_{a}d\eta_{b}},x\in \left\{a,b\right\}.$$

where Ω is the domain in which the signals are selected. The simplest way to describe such a domain is,

(5) $$\Omega^{square}=\left\{\left(\eta_{a},\eta_{b}\right)|\eta_{T_{A}}\leq \eta_{a}\leq 1,\eta_{T_{B}}\leq \eta_{b}\leq 1\right\}.$$

### 2.2. Free-Space SNS TF-QKD Based on Asymmetric Transmittance

Considering the fact that the users can only obtain the corresponding counts and error counts and use them to calculate the overall gain and quantum bit-error rate throughout the whole experiment and the two channels’ transmittances are asymmetric, the observable model proposed in [33] was adopted to calculate the SKR of the free-space SNS TF-QKD, that is:

(6) $$\begin{array}{cc} \; & R_{SNS}^{Observable}=R\left[\langle Q^{Z}\rangle ,\langle EQ^{Z}\rangle ,\langle Q_{d}^{X}\rangle ,\langle EQ_{d}^{X}\rangle \right],\\ \; & \langle Q\rangle =\frac{\int \int_{\Omega }\left[Q\left(\eta_{a},\eta_{b}\right)\cdot p_{AB}\left(\eta_{a},\eta_{b}\right)\right]d\eta_{a}d\eta_{b}}{\int \int_{\Omega }p_{AB}\left(\eta_{a},\eta_{b}\right)d\eta_{a}d\eta_{b}},\\ \; & \langle EQ\rangle =\frac{\int \int_{\Omega }\left[EQ\left(\eta_{a},\eta_{b}\right)\cdot p_{AB}\left(\eta_{a},\eta_{b}\right)\right]d\eta_{a}d\eta_{b}}{\int \int_{\Omega }p_{AB}\left(\eta_{a},\eta_{b}\right)d\eta_{a}d\eta_{b}}. \end{array}$$

Here, the superscripts *Z* and *X* respectively represent the Z-window (both users choose a signal window) and the X-window (both users choose a decoy window with other conditions met) in the SNS TF-QKD. The subscript d∈{v,w,0} denotes the intensities of light that they choose in different X-windows.

It should be noted that since the transmittances of the two channels’ are not the same in most cases, the method of calculating the original SNS TF-QKD’s SKR [20] is no longer suitable. Since the only asymmetric parameters considered in this work are channel transmittances, some simplifications could be adopted in the formulas for SKR [22]. Under this constraint, the Z-windows’ overall gains and quantum-bit errors can be written as the functions of $\eta_{a}$ and $\eta_{b}$ when other parameters are fixed,

(7) $$\begin{array}{cc} Q^{Z}\left(\eta_{a},\eta_{b}\right) & ={\left(1-\epsilon \right)}^{2}Y_{0}+2\epsilon^{2}\left(1-P_{d}\right)e^{-\frac{\eta_{a}+\eta_{b}}{2}\mu }\cdot \left[1-\left(1-P_{d}\right)e^{-\frac{\eta_{a}+\eta_{b}}{2}\mu }\right]\\ \; & +2\epsilon \left(1-\epsilon \right)\left(1-P_{d}\right)\left\{e^{-\frac{\eta_{a}}{2}\mu }\left[1-\left(1-P_{d}\right)e^{-\frac{\eta_{a}}{2}\mu }\right]+e^{-\frac{\eta_{b}}{2}\mu }\left[1-\left(1-P_{d}\right)e^{-\frac{\eta_{b}}{2}\mu }\right]\right\},\\ EQ^{Z}\left(\eta_{a},\eta_{b}\right) & ={\left(1-\epsilon \right)}^{2}Y_{0}+2\epsilon^{2}\left(1-P_{d}\right)e^{-\frac{\eta_{a}+\eta_{b}}{2}\mu }\cdot \left[1-\left(1-P_{d}\right)e^{-\frac{\eta_{a}+\eta_{b}}{2}\mu }\right], \end{array}$$

where $Y_{0}=2P_{d}\left(1-P_{d}\right)$ is the yield of the zero-photon, $P_{d}$ is the dark count rate, and ϵ is the probability of a user sending the signal state in a Z-window.

In the SNS TF-QKD protocol, each user should add a random continuous phase $\delta_{a}$($\delta_{b}$) to their states. If the conditions below are satisfied in the same round: (1) both users determine a decoy window; (2) their chosen intensities happen to be the same; (3) their added phases meet the criterion during the post-selection: $|\delta_{a}-\delta_{b}-m\pi |\leq \frac{2\pi }{M}$, where m=0,1 and *M* is the number of phase slices the users predetermine to divide the phase interval [0,2π) into; (4) only one detector clicks, then that time window is called an effective X-window, and the formulas of its corresponding overall gain and quantum-bit error can be listed as functions of four parameters,

(8) $$\begin{array}{cc} Q_{d}^{X}\left(\eta_{a},\eta_{b},\delta_{a},\delta_{b}\right) & =\left(1-P_{d}\right)e^{-\frac{\eta_{a}+\eta_{b}}{2}d}\left[e^{-cos\left(\delta_{a}-\delta_{b}\right)\sqrt{\eta_{a}\eta_{b}}d}+e^{cos\left(\delta_{a}-\delta_{b}\right)\sqrt{\eta_{a}\eta_{b}}d}\right]\\ \; & -2{\left(1-P_{d}\right)}^{2}e^{-(\eta_{a}+\eta_{b})d},\\ EQ_{d}^{X}\left(\eta_{a},\eta_{b},\delta_{a},\delta_{b}\right) & =\left(1-P_{d}\right)e^{-\frac{\eta_{a}+\eta_{b}}{2}d-cos\left(\delta_{a}-\delta_{b}\right)\sqrt{\eta_{a}\eta_{b}}d}-2{\left(1-P_{d}\right)}^{2}e^{-(\eta_{a}+\eta_{b})d}. \end{array}$$

Since both $\delta_{a}$ and $\delta_{b}$ are continuous, the probability of their difference strictly equaling zero or π will tend to zero, and this situation together with the asymmetric channel transmittances and the optical system’s misalignment error $E_{d}$ will cause an optical system error $\frac{1-cos(\Delta )}{2}$ [23]. Furthermore, such an error can be equivalently considered as the consequence of an extra phase difference $\Delta =arccos\left(1-2E_{sys}\right)=\frac{\sqrt{\eta_{a}\eta_{b}}d}{(\eta_{a}+\eta_{b})d}\left(1-2E_{d}\right)$ [22]. Furthermore, as can be seen, the above equations can be treated as functions of $\delta_{a}$ and $\delta_{b}$. One more step of integrating is needed to obtain the average overall gains and quantum-bit errors,

(9) $$\begin{array}{cc} \; & Q_{d}^{X}\left(\eta_{a},\eta_{b}\right)=\frac{M^{2}}{4\pi^{2}}\int_{\Delta }^{\Delta +\frac{2\pi }{M}}\int_{0}^{\frac{2\pi }{M}}Q_{d}^{X}\left(\eta_{a},\eta_{b},\delta_{a},\delta_{b}\right)d\delta_{a}d\delta_{b},\\ \; & EQ_{d}^{X}\left(\eta_{a},\eta_{b}\right)=\frac{M^{2}}{4\pi^{2}}\int_{\Delta }^{\Delta +\frac{2\pi }{M}}\int_{0}^{\frac{2\pi }{M}}EQ_{d}^{X}\left(\eta_{a},\eta_{b},\delta_{a},\delta_{b}\right)d\delta_{a}d\delta_{b}. \end{array}$$

Now that the X-windows’ and Z-windows’ overall gains and quantum-bit errors have been introduced, the model mentioned above can finally be applied to calculate these parameters in the free space with and without the P-RST method. After all these been performed, the SKR can be calculated as [20],

(10) $$\begin{array}{cc} \; & R_{SNS}^{Observable}\left(\Omega \right)=\int \int_{\Omega }R\left[\langle Q^{Z}\rangle ,\langle EQ^{Z}\rangle ,\langle Q_{d}^{X}\rangle ,\langle EQ_{d}^{X}\rangle \right]\cdot p_{AB}\left(\eta_{a},\eta_{b}\right)d\eta_{a}d\eta_{b}\\ \; & R=2\epsilon \left(1-\epsilon \right)\mu e^{-\mu }Y_{1}^{L}\left[1-H\left(e_{1}^{U}\right)\right]-fQ^{Z}H\left(E^{Z}\right), \end{array}$$

where $E^{Z}=\frac{EQ^{Z}}{Q^{Z}}$ is the Z-windows’ quantum bit-error rate, *f* is the error correction efficiency factor, $H\left(x\right)=-xlog_{2}\left(x\right)-\left(1-x\right)log_{2}\left(1-x\right)$ is the binary entropy, and $Y_{1}^{L}$ and $e_{1}^{U}$ are respectively the lower and upper bound of the single-photon’s yield and quantum bit-error rate, which can be calculated following a common decoy-state method [20]. Since the observable model should be applied, one should obtain $\langle Q^{Z}\rangle$, $\langle EQ^{Z}\rangle$, $\langle Q^{X}\rangle$ and $\langle EQ^{X}\rangle$ with Equation (6) and then turn to Equation (10) for the free-space SNS TF-QKD’s SKR with these expectation values.

Note that the normal steps of post-selecting signals based on transmittance are setting a threshold, monitoring each rounds’ real-time channel transmittances, and discarding those less than the threshold. This can be obtained by numerical optimization when the experiment is performed [30,31]. However, the P-RTS method demonstrates a different way. It was firstly proposed in [32]. Due to the fact that the selection threshold is independent of the PDTC and can only be determined by the experimental parameters, the threshold is able to be predetermined before the experiment. Thus, determining the threshold will be the first thing to perform in the next section after plotting out $R(\eta_{a},\eta_{b})$.

## 3. Numerical Simulation

The letter Ω in Equation (6) denotes the area, a 2D threshold for the signals’ selection. In order to obtain this area, the key rate function $R(\eta_{a},\eta_{b})$ should be firstly plotted out. It should be noted that this key rate function is only determined by the signals’ intensities and experimental parameters. We list the experimental parameters such as misalignment error rate, dark count rate, etc., in Table 1. The signals’ intensities and the probability of the signals being sent or not are given as u=0.4, v=0.01, w=0.005, and ϵ=0.02, and only the infinite data size was considered.

**Figure 1:**
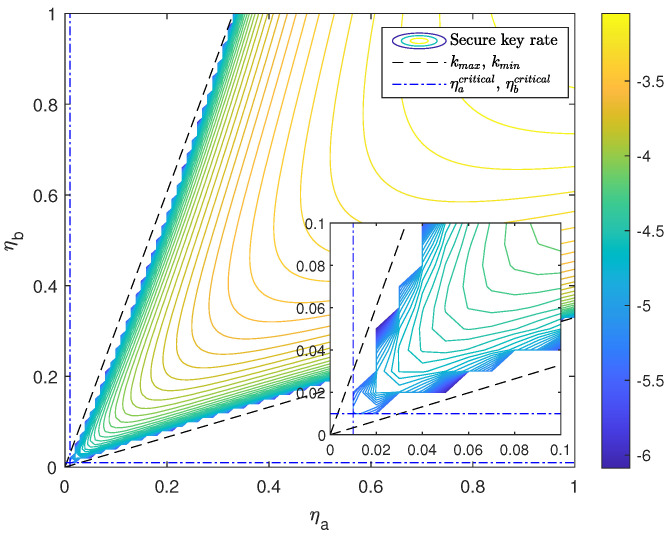
The SNS TF-QKD’s key rate function $R(\eta_{a},\eta_{b})$ with asymptotic lines, $x_{min}$ and $x_{max}$, and horizontal/vertical tangents, $\eta_{a}^{critical}$ and $\eta_{b}^{critical}$, of the R=0 contour.

Figure 1 shows the key rate function of the free-space SNS TF-QKD with asymptotic lines. In Figure 1, the contour, the two asymptotic lines, and the horizontal/vertical tangents of the R=0 contour respectively represent the SKR, the minimum and maximum channel transmittance mismatch $k=\frac{\eta_{b}}{\eta_{a}}$ that can be reached, and the minimum channel transmittances.

Clearly, the most suitable area of the signals’ selection is the domain of Figure 1 where the condition $R(\eta_{a},\eta_{b})\geq 0$ is satisfied,

(11) $$\Omega^{Selected}=\left\{\left(\eta_{a},\eta_{b}\right)|R\left(\eta_{a},\eta_{b}\right)\geq 0\right\}.$$

However, it may be difficult to directly apply the domain above to the double-integral for the 2D threshold of signals’ selection. For simplicity, a replaced threshold domain, which can play an approximate role in the double-integral, is proposed [33]. The replaced domain is formed by the four lines in Figure 1 and is similar to $\Omega^{Selected}$,

(12) $$\Omega^{Similar}=\left\{\left(\eta_{a},\eta_{b}\right)|\eta_{a}^{critical}\leq \eta_{a}\leq 1,\eta_{b}^{critical}\leq \eta_{b}\leq 1,k_{min}\leq \frac{\eta_{b}}{\eta_{a}}\leq k_{max}\right\},$$

where $k_{min}$ and $k_{max}$ are the slope of $x_{min}$ and $x_{max}$, respectively.

Since the function $R(\eta_{a},\eta_{b})$ only depends on the experimental parameters and the signals’ intensities, the four lines $x_{min}$, $x_{max}$, $\eta_{a}^{critical}$, and $\eta_{b}^{critical}$ are all independent of the PDTC of the channels. Thus, this 2D threshold formed by these four lines can be predetermined even before the experiment.

**Figure 2:**
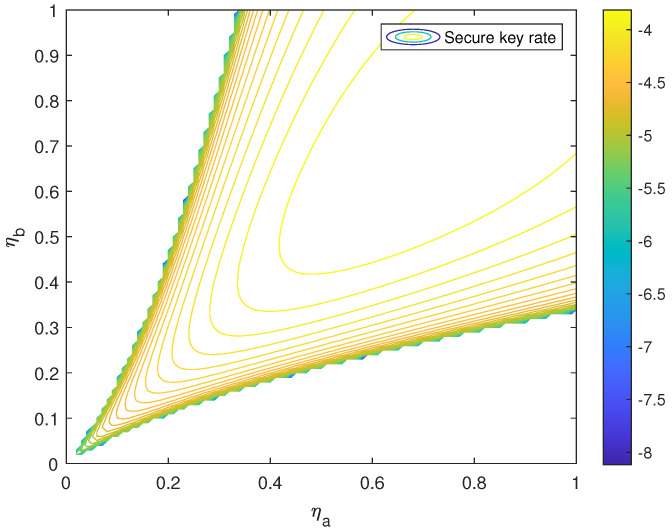
$R(\eta_{a},\eta_{b})$ without the P-RTS method based on the observable model.

**Figure 3:**
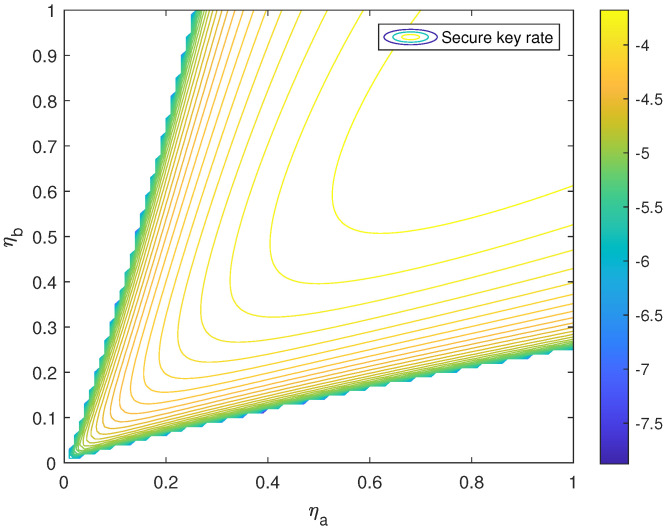
$R(\eta_{a},\eta_{b})$ with the P-RTS method based on the observable model.

Here, in order to demonstrate the effect of the P-RTS method, the SKR function $R(\eta_{a},\eta_{b})$ of the free-space SNS TF-QKD with and without the P-RTS method based on the observable model is plotted out in Figure 2 and Figure 3. The parameters in Table 1 are also used in the same way. The variance of the PDTC was set to 0.75. Obviously, the near-hyperbolic R=0 contour of the function $R(\eta_{a},\eta_{b})$ without the P-RTS is narrower than that of the function with the P-RTS. In other words, the area of $R(\eta_{a},\eta_{b})\geq 0$ with the P-RTS method becomes larger, so that the protocol can reach a larger channel transmittance mismatch.

**Figure 4:**
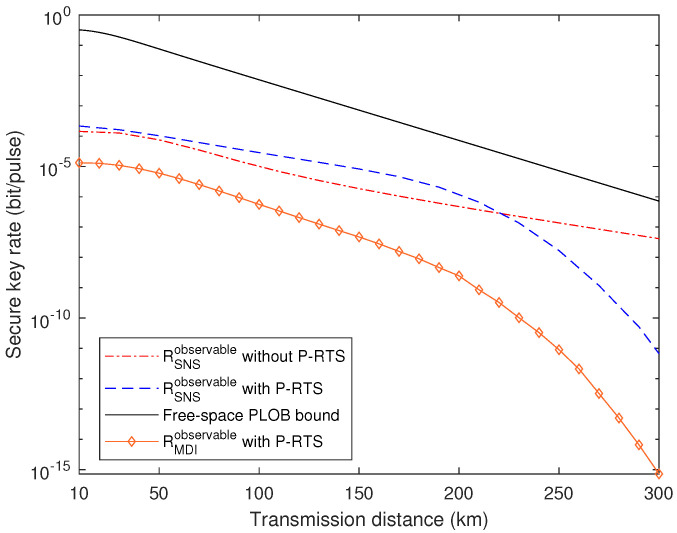
Comparison of the free-space SNS TF-QKD with and without the P-RTS method based on the observable model and the free-space PLOB bound versus the transmission distance.

In order to vividly compare the SKR performance of the free-space SNS TF-QKD with and without the P-RTS, we further plotted out the functions versus total transmission distance, i.e., sum of $L_{AC}$ (channel length between Alice and Charlie) and $L_{BC}$ (channel length between Bob and Charlie), with $L_{AC}=L_{BC}+10$, in Figure 4. The parameters in Table 1 were also used in the same way. The PDTC’s variance was set to 0.75, meaning AT is quite strong. The channel transmission loss rate was assumed to be the same as that of optical fiber, α = 0.2 dB/km. The black solid line denotes the PLOB bound in the free space. Based on the ratewise integration model in [32] and Equation (26) in [36], we used Equation (13) as the formula for calculating the PLOB bound in the free space with a certain mean transmittance $\eta_{0}$, which corresponds to a certain transmission distance,

(13) $$K=\int_{0}^{1}-log_{2}\left(1-\eta \cdot \eta_{d}\right)\cdot p_{\eta_{0},\sigma }\left(\eta \right)d\eta .$$

As can be seen in Figure 4, $R_{SNS}^{observable}$ with the P-RTS has better performance than that without the method when the transmission distance is below around 220 km. The former one drops more sharply than the latter one after 190 km. The reason for this should be that after a certain point, as the channel transmittance increases with the transmission distance, it becomes more difficult to find the experiment round that satisfies the condition of the P-RTS, making the SKR decrease more quickly. Meanwhile, neither of these two can surpass the free-space PLOB bound. The reasons may be that the atmospheric turbulence’s effect on the SNS TF-QKD is very large or this free-space PLOB bound we evaluated is not tight enough. The solid line with diamond symbols represents the MDI-QKD with the P-RTS with the misalignment error $e_{d}=0.075$ since $e_{d}=0.15$ in Table 1 is intolerable for the protocol. One can see that its performance is not as good as that of the SNS-TF QKD with and without the P-RTS.

## 4. Conclusions

In the paper, we presented a prefixed-threshold selection for the free-space SNS TF-QKD, where we demonstrated the more accurate observable model for the free-space channel to calculate the SKR in the case of AT. Based on the contour of the SKR function $R(\eta_{a},\eta_{b})$, we can predetermine the 2D threshold for the signals’ selection. The numerical simulation results showed that it is possible to share a secure key by using the free-space SNS TF-QKD. The results also showed that the free-space SNS QKD protocol with the P-RTS can decrease the influence caused by AT at a short distance, and the performance of the protocol with and without the P-RTS is better than the MDI-QKD with the method, even with larger misalignment error. Thus, our work has some benefits for the free-space SNS TF-QKD in practice.

## Figures and Tables

**Table 1:** The parameters of the numerical simulation.

Parameters	$p_{d}$	$e_{0}$	$e_{d}$	$\eta_{d}$	α	*f*
**Value**	$1\times 10^{-10}$	0.5	0.15	0.5	0.2	1.1

## Data Availability

Not applicable.

## Abbreviations

The following abbreviations are used in this manuscript:

QKD	Quantum key distribution
TF-QKD	Twin-field quantum key distribution
SNS TF-QKD	Sending-or-not twin-field quantum key distribution
MDI-QKD	Measurement-device-independent quantum key distribution
RRDPS-QKD	Round-robin differential-phase-shift quantum key distribution
AT	Atmospheric turbulence
P-RTS	Prefixed-threshold real-time selection
PDTC	Probability distribution of the transmission coefficient
